# Adropin as a potential marker of enzyme-positive acute coronary syndrome

**DOI:** 10.5830/CVJA-2016-055

**Published:** 2017

**Authors:** Suna Aydin, Mehmet Nesimi Eren, Musa Yilmaz, Meltem Yardim, Suleyman Aydin, Mehmet Kalayci, Omer Dogan Alatas, Tuncay Kuloglu, Heseyin Balaban, Tolga Cakmak, Mehmet Ali Kobalt, Ahmet Çelik

**Affiliations:** Department of Anatomy – Cardiovascular Surgery, Elazig Education and Research Hospital, Elazig, Turkey; Department of Cardiovascular Surgery, School of Medicine, Dicle University, Diyarbakir, Turkey; Department of Medical Biochemistry (Firat Hormones Research Group), School of Medicine, Firat University, Elazig, Turkey; Department of Medical Biochemistry (Firat Hormones Research Group), School of Medicine, Firat University, Elazig, Turkey; Department of Medical Biochemistry (Firat Hormones Research Group), School of Medicine, Firat University, Elazig, Turkey; Laboratory of Medical Biochemistry, Elazig Education and Research Hospital, Elazig, Turkey; Department of Emergency, Mugla Sitki Kocman University, Education and Research Hospital, Mugla 48000, Turkey; Department of Histology and Embryology, School of Medicine, Firat University, Elazig, Turkey; Department of Internal Medicine, 29 May State Hospital, Ankara, Turkey; Department of Cardiology, Ercis State Hospital, Van, Turkey; Department of Cardiology, School of Medicine, Firat University, Elazig, Turkey; Department of Cardiology, School of Medicine, Mersin University, Mersin, Turkey

**Keywords:** saliva, serum, adropin, acute coronary syndrome, enzyme-positive acute coronary syndrome, myocardial infarction, immunohistochemistry

## Abstract

**Aim:**

Enzyme-positive acute coronary syndrome (EPACS) can cause injury to or death of the heart muscle owing to prolonged ischaemia. Recent research has indicated that in addition to liver and brain cells, cardiomyocytes also produce adropin. We hypothesised that adropin is released into the bloodstream during myocardial injury caused by acute coronary syndrome (ACS), so serum and saliva levels rise as the myocytes die. Therefore, it could be useful to investigate how ACS affects the timing and significance of adropin release in human subjects

**Methods:**

Samples were taken over three days after admission, from 22 EPACS patients and 24 age- and gendermatched controls. The three major salivary glands (submandibular, sublingual and parotid) were immunohistochemically screened for adropin production, and serum and saliva adropin levels were measured by an enzyme-linked immunosorbent assay (ELISA). Salivary gland cells produce and secrete adropin locally.

**Results:**

Serum adropin, troponin I, CK and CK-MB concentrations in the EPACS group became gradually higher than those in the control group up to six hours (p < 0.05), and troponin I continued to rise up to 12 hours after EPACS. The same relative increase in adropin level was observed in the saliva. Troponin I, CK and CK-MB levels started to decrease after 12 hours, while saliva and serum adropin levels started to decrease at six hours after EPACS. In samples taken four hours after EPACS, when the serum adropin value averaged 4.43 ng/ml, the receiver operating characteristic curve showed that the serum adropin concentration indicated EPACS with 91.7% sensitivity and 50% specificity, while when the cut-off adropin value in saliva was 4.12 ng/ml, the saliva adropin concentration indicated EPACS with 91.7% sensitivity and 57% specificity.

**Conclusion:**

In addition to cardiac troponin and CK-MB assays, measurement of adropin level in saliva and serum samples is a potential marker for diagnosing EPACS.

## Aim

Acute coronary syndrome (ACS) [acute myocardial infarction (AMI), enzyme-positive acute coronary syndrome (EPACS)] is the dominant cause of death and disability in children and in young,^1^ middle-aged and elderly adults in both developed and developing countries.^2^ Coronary arteriosclerosis is a chronic disease with stable and unstable periods.^3^ During unstable periods, increased cholesterol deposition and activated local inflammation in the vascular wall can cause atheromatous plaque rupture and thrombus formation, resulting in unstable angina (chest pain) or MI (heart attack).^4^,^5^

EPACS is currently diagnosed, according to criteria proposed by the American College of Cardiology (ACC) and European Society of Cardiology (ESC),^06^,^07^ as the presence of three or more of the following abnormalities: a history of the presenting illness, prolonged chest pain, ‘silent infarct’, pathological Q waves in the electrocardiogram (ECG), typical rise and/or fall of cardiac biomarkers (preferably troponin I) with at least one value above the 99th percentile of the upper reference limits.^08^

Despite this abundance of parameters for diagnosing EPACS, a million patients annually seek care in emergency, cardiology and cardiovascular surgery departments with chest pain or other symptoms suggesting an ACS, although only around 10% are subsequently confirmed to have EPACS.^9^ Therefore, emergency, cardiology and cardiovascular surgery doctors need novel advance, accurate, fast, easily accessible and cost-effective cardiac markers for better patient outcomes and fewer complications.

Adropin is a peptide hormone secreted from pancreatic, liver, brain and kidney tissues and from the endocardium, myocardium and epicardium of the heart.^10^-^12^ It circulates in the blood to activate the release of nitric oxide and regulate apoptosis and energy homeostasis,^13^,^14^ and could be a novel predictor of heart failure. Adropin secretion is controlled by many factors including glucose levels and myocardial infarction.^10^

Decreased adropin level is an independent risk factor for endothelial dysfunction, a key early event in atherogenesis, and is integral to the onset of coronary artery disease (CAD) and ACS.^15^ It is also an independent predictor of clinically relevant coronary atherosclerosis.^16^ Adropin levels are significantly lower in patients with cardiac syndrome X than in healthy subjects, so low serum adropin level could be an independent risk factor for this condition.^15^ It is also closely related totype 2 diabetes mellitus and gestational diabetes mellitus.^16^,^17^ In addition, a recent study revealed that adropin levels were decreased in patients with late saphenous vein graft occlusion and it could have been causally related.^18^

On the basis of these findings, it was hypothesised that the adropin synthesised in the endocardium, myocardium and epicardium^10^ could serve as a novel biological marker for the diagnosis and prognosis of myocardial ischaemia, because ischaemic injury to heart muscle cells is likely to release adropin into the bloodstream. However, there have been contradictory reports from animal studies that examined the association between adropin expression and isoproterenolinduced myocardial infarction, which indicated that the gradual increase in serum adropin levels could serve as an alternative to troponin I measurement for diagnosing EPACS,^19^ and human studies, showing that single-timing serum adropin levels were lower in EPACS patients than in stable angina pectoris (SAP) patients or controls.^20^

This conflict needs to be resolved. Therefore, the purposes of this study were: (1) to determine the changes in adropin and troponin I concentrations in sera from EPACS patients; (2) to determine whether this hormone is produced by the three major salivary glands, parotid, sublingual and submandibular; and (3) to determine whether saliva contains adropin, because obtaining saliva samples is non-invasive, making it advantageous over blood sampling.

## Methods

All protocols for the human studies in this work accorded with the principles set out (date 6/3/2014; issue no: 03) by the Institutional Human Ethics Committee (FUIHC) and with the ethical principles in the most recent version of the Declaration of Helsinki. Written informed consent to participate in the study was individually obtained.

A total of 46 subjects (22 EPACS patients and 24 controls) were admitted to the Emergency Department at Elazig Education and Research Hospital due to chest pain or other symptoms (within 30–40 minutes of onset). Our hospital is conveniently located in downtown Elazig so it can be reached from any part of the city within 15 minutes of the first symptoms. The heart team (cardiologists and cardiovascular surgeons) evaluated the patients admitted, as described previously.^21^

A diagnosis of EPACS was made by integrating the history of the presenting illness, an increase in serum troponin I concentration (1 × upper limit of the hospital normal range), and associated symptoms of ischaemia, chest pain and/or characteristic ECG signs (ST-segment–T-wave changes or development of pathological Q waves).^6^-^8^ All patients (n = 22) were screened for EPACS by coronary angiography. Healthy volunteers (n = 24) having routine annual check ups (08.00– 09.00) served as controls.

The patients were treated as explained elsewhere.^21^ Briefly, their EPACS was treated as primary PCI (first loading dose 600 mg clopidogrel + 300 mg acetylsalicylic acid/day, and maintained on 75 mg clopidogrel + 300 mg acetylsalicylic acid/day; n = 5), thrombolytic therapy was given (10 U reteplase + 75 mg clopidogrel + 300 mg acetylsalicylic acid/day; n = 9), and routine anti-anginal therapy was provided (first loading dose 600 mg clopidogrel + 300 mg acetylsalicylic acid/day, and maintained on 75 mg clopidogrel + 300 mg acetylsalicylic acid/day; n = 8).

Exclusion criteria were: over 75 or under 50 years old, surgery or trauma within two months of the study, known cardiomyopathy, and family history of cardiovascular disease (CVD) (having a father who developed CVD before 55 years of age, a mother before 65 years, or a sibling at any age).

We defined CVD as coronary heart disease, hypertension (hypertension was defined as resting systolic blood pressure (SBP) ≥ 140 mmHg and/or diastolic blood pressure (DBP) ≥ 90 mmHg according to WHO–ISH criteria),22 or on current antihypertensive treatment, rheumatic heart disease, known malignant diseases, febrile conditions, acute or chronic inflammatory disease, gastrointestinal diseases, suspected myocarditis or pericarditis, diabetes mellitus of any type, severe heart failure, advanced renal or hepatic disease, alcohol consumption of more than one unit per day, no regular intense exercise (> 15 min of aerobics three times per week), and use of tobacco products (former and current).

All the study participants, including the control subjects,underwent a standard clinical examination. Other details relevant to the EPACS studies were described previously.^8^,^21^

The first saliva and venous blood samples were collected when patients were admitted to the Emergency Department (within 30–40 minutes of onset) and before angiography. Other samples (two, four, six, 12, 24, 48 and 72 hours) were drawn from the antecubital veins of all participants into plain sterile tubes for serum, and into sterile urine cups for whole resting saliva at 08.00 hours in the Department of Cardiology. Saliva and serum were collected simultaneously at each sampling time after thorough rinsing of the mouth with water, as previously described.^21^,^23^,^24^

Circadian variation in the onset of EPACS has been documented. To avoid this influence, only EPACS patients admitted in the morning were included in this study.

Blood samples were divided into two aliquots, one for classical biochemical parameters and the other for measuring adropin levels. The plain sterile tubes for blood and sterile urine cups for saliva contained 500 kIU aprotonin to preclude proteolysis, and after clotting, the samples, were immediately centrifuged at 4 000 rpm and kept frozen (−80°C) pending analysis.

All samples were subjected to conventional laboratory analyses using an autoanalyser, including determination of glucose, total cholesterol (TC), triglyceride (TG), low-density lipoprotein cholesterol (LDL-C) and high-density lipoprotein cholesterol (HDL-C) concentrations. Serum troponin I concentration was measured by chemiluminescence using a Siemens IMMULITE 2000 XPi immunoassay system (Siemens Healthcare Diagnostics Inc, Flanders NJ, USA) and commercial kits (Siemens Healthcare Diagnostics Products Ltd, Llanberis, United Kingdom).

Serum and saliva adropin levels were measured using the same commercial EIA kits (cat no: EK-032-35) and procedures (Phoenix Pharmaceuticals, Belmont, CA, USA). The saliva adropin assay was validated according to previously published methods.^25^ The lowest detectable concentration of adropin was 0.01 ng/ml, with intra- and inter-assay variations of 10 and 15%, respectively. Absorbance at 450 nm was measured with an ELX 800 ELISA reader.

Salivary glands were obtained from the Department of Pathology. They had been removed by surgeons only because of calcification. Adropin was screened immunohistochemically using the Hsu et al. avidin–biotin peroxidase complex (ABC) method, as recently described.^26^ Adropin primary antibody was diluted 1/200 (rabbit polyclonal anti-adropin antibody, ab12800; Abcam, Cambridge, UK), applied and incubated for 60 min in a humid chamber at room temperature.

Immunostained sections from the parotid, submandibular and sublingual glands were examined with an Olympus BX 50 photomicroscope. Immunohistochemical staining was scored for both intensity and prevalence on a scale of 0 to +3 (0: absent, +1: weak, +2: medium, +3: strong).

## Statistical analysis

All statistical analyses were performed using SPSS for Windows version 21.0 (SPSS Inc, Chicago, USA). Differences between groups were analysed with the Kruskal–Wallis test. The Mann–Whitney U-test was used to compare parameters within groups. Comparisons of mean values between groups were expressed as ± 2 SEM.

The correlation between serum adropin levels and other clinical characteristics in EPACS patients was measured as the Spearman correlation coefficient, or a chi-squared value when appropriate. Probability values were considered significant at p < 0.05.

## Results

**Fig. 1.**
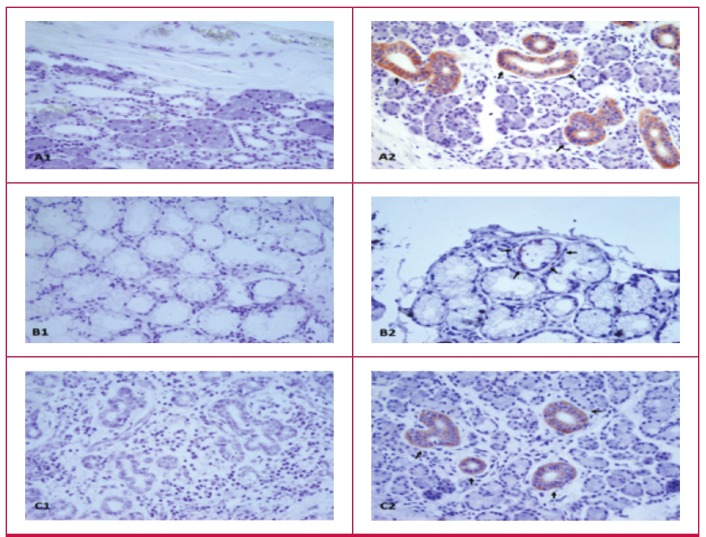
Adropin immunohistochemistry of the intercalated duct of the parotid, striated and interlobular ducts of the submandibular, and mucous acinus of the sublingual glands. A1, parotid negative; A2: parotid adropin immunoreactivity; B1, sublingual negative; B2: sublingual adropin immunoreactivity, C1, submandibular negative; C2, submandibular adropin immunoreactivity. Red colour shows adropin immunoreactivity. Magnification ×400.

Immunohistochemical studies revealed no adropin immunoreactivity in the negative controls (secondary antibody omitted or phosphate-buffered saline used) for the parotid (Fig. 1A1), sublingual (Fig. 1B1) and submandibular (Fig. 1C1) glands, but when the adropin antibody was used, there was reactivity (red colour) in all three salivary glands (Fig. 1A2, intercalated duct immunoreactive to adropin antibody; 1Fig. 1B2, mucous acinus immunoreactive to adropin antibody; Fig.1C2, striated duct, interlobular duct immunoreactive to adropin antibody) as distinguished histologically.

Table 1 shows the differences in glucose and lipid profiles (TC, HDL-C, LDL-C and TG) in subjects with and without EPACS. Glucose levels in EPACS patients were higher than in the controls but still within the normal range. Lipid profiles were not affected by EPACS (Table 1).

**Table 1:** Changes in glucose and lipid profiles with and without EPA CS. All values are presented as mean ± SD .

*Parameters*	*Control (n = 24)*	*EPACS (n = 22)*	*p-value*
Age (years)	40.57 ± 5.0	57.75 ± 6.38	0.000
Male/female	12/12	12/10	0.713
Glucose (mg/dl)	89.07 ± 6.27	102.08 ± 19.24	0.039
(mmol/l)	(4.94 ± 0.35)	(5.67 ± 1.07)	
Total cholesterol (mg/dl)	178.93 ± 42.82	212.13 ± 44.82	0.111
(mmol/l)	(4.63 ± 1.11)	(5.49 ± 1.16)	
HDL-C (mg/dl)	43.42 ± 9.2	40.4 ± 7.06	0.535
(mmol/l)	(1.12 ± 0.24)	(1.05 ± 0.18)	
LDL-C (mg/dl)	106.14 ± 31.69	130.25 ± 37.65	0.105
(mmol/l)	(2.75 ± 0.82)	(3.37 ± 0.98)	
Triglycerides (mg/dl)	172.93 ± 57.37	200.33 ± 86.16	0.354
(mmol/l)	(1.95 ± 0.65)	(2.26 ± 0.97)	

Validation of the EIA kit (cat no: EK-032-35) showed it to be as sensitive to saliva adropin as to serum adropin. The lowest detectable adropin concentration in saliva was 0.01 ng/ml, with intra-assay (within day) and inter-assay (between days) variations of less than 10 and 12%, respectively. Assay recovery was between 98 and 106%. The response to salivary adropin was linear over the range 0.50–16.5 ng/ml. Therefore, the sensitivity and specificity of the EIA kit were the same for saliva as for serum adropin concentrations.

**Fig. 2.**
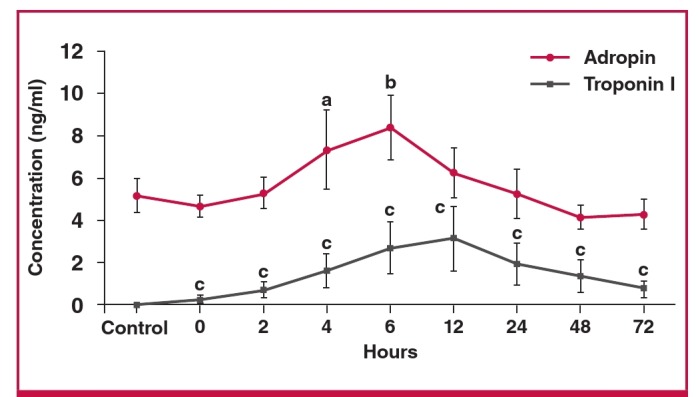
Differences in serum adropin and troponin I concentrations between EPACS and control subjects. ap < 0.05 and b,cp < 0.01 compared with control.

The serum adropin concentration was slightly (insignificantly) lower in samples taken within 30 minutes (zero time) of patient admission, than the corresponding control value and stable coronary diseases (0.67–0.8 ng/ml; n = 9). It rose within two hours post infarct and peaked at six hours; the adropin concentrations at four and six hours post infarct were significantly higher than the controls. At 12 and 24 hours post infarct, the levels were also higher than the corresponding control values but not significantly so (Fig. 2).

The serum troponin I concentration rose within 30 minutes (zero time: blood taken immediately after the patient was admitted to hospital), peaked at 12 hours post infarct, and remained significantly higher than the controls for up to 72 hours (Fig. 2). The time course of changes in adropin level paralleled that of troponin I. These findings demonstrate that serum adropin might help, in conjunction with troponin levels, in the early diagnosis of EPACS.

**Fig. 3.**
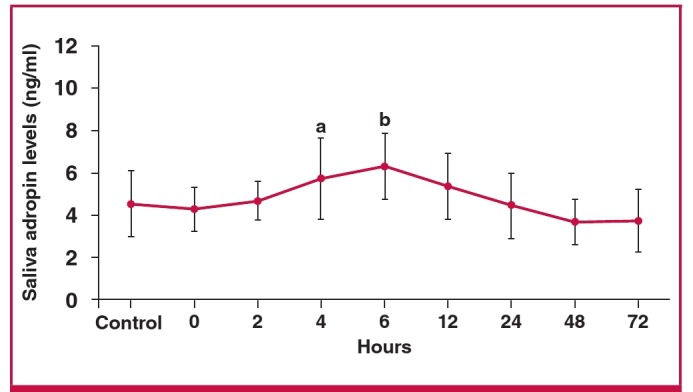
Differences in saliva adropin concentrations between EPACS and control subjects. ap < 0.05 and bp < 0.01 compared with control.

The saliva adropin concentration was also slightly lower than the controls (not statistically significant) in samples taken within 30 minutes (zero time) of hospital admission. Like the serum adropin concentration, saliva adropin then rose within two hours post infarct and peaked at six hours, being significantly higher than the controls at four and six hours, and remaining elevated at 12 and 24 hours post infarct (Fig. 3). These results show that serum and saliva adropin concentrations increased and decreased in parallel in EPACS patients.

Serum adropin levels were positively correlated with saliva adropin levels (r = 0.763, p ≤ 0.01) but with neither glucose level nor lipid profiles. There was also a correlation between serum adropin and cTnT levels (r = 0.68, p = 0.000). Therefore, measuring saliva adropin levels may be an alternative to measuring serum adropin concentrations for diagnosing EPACS or metabolic diseases, for example, diabetes, in which adropin regulates energy homeostasis and insulin resistance.^27^

**Fig. 4:**
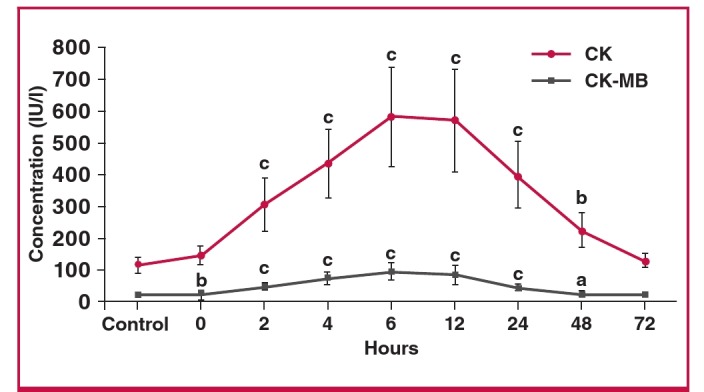
Differences in serum CK and CK-MB concentrations between EPACS and control subjects. ap < 0.05 and b,cp < 0.01 compared with control.

Serum CK and CK-MB levels were also measured. The initial statistically significant rise in CK-MB above control concentrations occurred within 30–40 minutes (zero time) after the onset of chest pain, peaked at six hours, and returned to baseline at 72 hours. CK levels also started to increase within 30–40 minutes after EPACS (zero time sample: blood taken immediately on admission) and peaked at six hours, thereafter decreasing up to 72 hours. The concentration of CK was significantly higher than the controls from two hours up to 48 hours in the EPACS patients (Fig. 4).

**Fig. 5.**
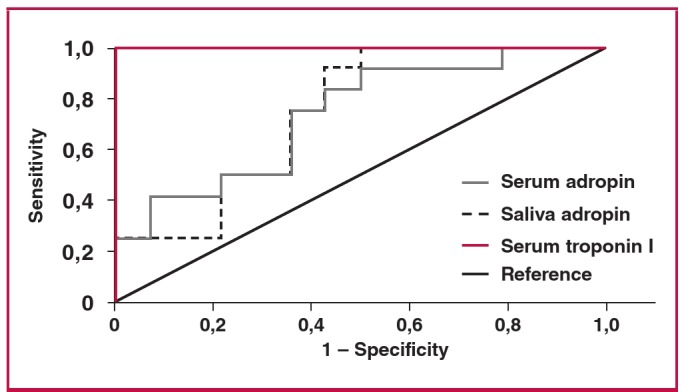
Sensitivity and specificity of serum and saliva adropin and serum troponin I for detecting EPACS at four hours. The area under the ROC curve, adropin sensitivity of 91.7% and specificity of 67%, were identified when the cut-off was set at 5.37 ng/ml adropin.

**Fig. 6.**
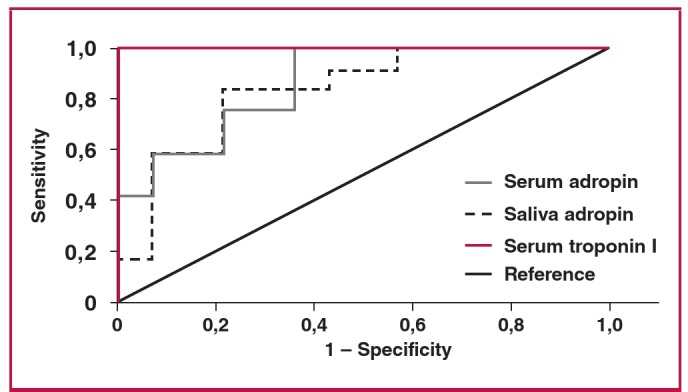
Sensitivity and specificity of serum and saliva adropin and serum troponin I for detecting EPACS at six hours. The area under the ROC curve, adropin sensitivity of 91.7% and specificity of 50%, were identified when the cut-off was set at 4.43 ng/ml adropin.

At four hours after EPACS, the serum adropin concentration measurement had a sensitivity of 91.7% and specificity of 50% at a confidence interval of 95% when the cut-off value was set at 4.12 ng/ml (Fig. 5). At six hours after EPACS, with the cut-off value set to 5.37 ng/ml adropin for serum, the sensitivity was 91.7% and the specificity 64% (Fig. 6). At four hours after EPACS, saliva adropin exhibited 91.7% sensitivity and 57% specificity at a confidence interval of 95%, when the cut-off value was 4.12 ng/ml. At six hours after EPACS, when the cut-off value was 4.12 ng/ml, the same sensitivity and specificity were found as at four hours for saliva adropin level. Serum troponin I exhibited 100% sensitivity and 100% specificity at a confidence interval of 95% when the cut-off value was 0.141 ng/ml at four hours after EPACS, and when the cut-off value was 0.226 ng/ml at six hours after EPACS (Fig. 6).

## Discussion

Cardiovascular diseases are the leading cause of death in both developed and developing countries. The World Health Organisation predicts that by 2020, 37% of all deaths worldwide will be from CVDs.^22^ Health innovations are widely used, but ACS, a lethal manifestation of CVD, remains the leading cause of death worldwide.^28^ Currently, cardiac biomarkers (especially cTI and cTnT) are the most important diagnostic laboratory tests for ACS.8 Each year worldwide a million patients with suspected ACS are admitted to emergency, cardiology and cardiovascular surgery departments but only around 10% of cases are then confirmed.^9^ Therefore, an accurate, precise and rapid diagnostic test for EPACS is needed to save lives.

In this context, recent animal studies and a human study have suggested that adropin could be useful for diagnosing EPACS in addition to other cardiac biomarkers, but these studies were controversial.^19^,^20^ Therefore the ability of adropin to identify cardiac injury earlier than is possible with current biomarkers should be re-investigated and the controversy resolved. In the present study, therefore, we measured serum cardiac marker enzymes and timed serum and saliva adropin concentrations in EPACS patients and in age- and gender-matched controls.

Troponin I, CK, CK-MB and adropin concentrations gradually increased in the EPACS group from up to six hours to levels higher than in the controls (p < 0.05), and troponin I and CK-MB continued to increase for up to 12 hours after EPACS. After 12 hours, CK and adropin levels started to decrease for up to 72 hours. These findings confirmed the value of the classical parameters of troponin I, CK and CK-MB for diagnosing EPACS in clinical practice.

Saliva adropin concentrations changed in parallel with serum adropin concentrations in ACS. The saliva adropin concentration was generally higher than the serum adropin, possibly because the salivary glands produce adropin (see below). Since adropin is expressed in many tissues, including the heart, all contributing to the serum pool, we had assumed that serum adropin concentration increased after EPACS, as do troponin I or CK-MB, which are released from the myocardium, mainly during EPACS and necrosis following heart injury.^9^

Our clinical results agree with our previous animal experiments, showing that adropin concentration gradually rose above control levels in EPACS patients. This is in contrast to Yu et al., who found that serum adropin levels were significantly lower in EPACS patients than in SAP patients or controls.^20^ Yu et al. argued that their result indicates deficient adropin expression in EPACS patients, and adropin deficiency could be involved in the development and progression of EPACS. The reason for the difference in results is unknown.

Yu et al. measured single-time serum adropin levels in EPACS patients, while in our study we measured the time courses of serum and salivary adropin levels in patients and controls.^20^ In our zero-time samples, serum and saliva adropin values were slightly (insignificantly) lower in EPACS patients than in controls, and this could have corresponded to the single-time values measured by Yu et al.^20^ The adropin levels then started to increase and peaked at six hours after EPACS, potentially explaining the apparent conflict. Also, the mean adropin level is reported to be significantly lower in certain diseases, including in patients with late saphenous vein graft occlusion.^18^ Another possibiliy is that the adropin level was reduced in the baseline blood sample taken within 30 to 40 minutes of admission to hospital.

Although the glucose level was within normal physiological limits in the EPACS patients, it was higher than in the controls. This could have been due to the effect of increased epinephrine secretion after EPACS, causing glycogenolysis in the liver and releasing glucose into the blood.^29^,^30^ There was an inverse relationship between adropin and glucose levels.^10^,^12^,^16^,^17^ Yu et al. reported the same glucose levels. Different drugs used to treat EPACS could also have affected the adropin levels differently.20 Here we also assumed that the increased expression of adropin in saliva and serum could indicate acute cardiac injury caused by ACS, and could be central to the development of key pathologies associated with EPACS in humans, but further studies are needed to resolve the conflict between findings.

Salivary glands are now known to secrete a range of peptides/proteins involved in regulating endocrine metabolism.^21^ Therefore, in this study we also investigated whether the salivary glands produce adropin. The immunochemical findings indicated that adropin is one of the most abundant proteins secreted by human salivary glands, as previously described for peptides such as irisin,^21^ ghrelin^23^,^24^,^31^ and hepcidin.^32^ Adropin is synthesised in the intercalated duct of the parotid, the mucous acinus of the sublingual, and the striated and interlobular ducts of the submandibular glands, and is co-localised with irisin,^21^ ghrelin^23^,^24^,^31^ and hepcidin32 in those glands. Its expression has also been demonstrated in the liver, brain, cerebellum, kidneys, heart, pancreas and vascular tissues.10 The ELISA results in this study revealed that salivary and serum adropin levels were substantially higher in EPACS patients than in the controls and stable CAD patients (0.67–0.8 ng/ml).

The adropin levels in saliva were already elevated and increasd further at four and six hours after EPACS. The origin of the high salivary adropin levels is not known but it probably comes from the plasma after saturation, or a larger amount of cardiac adropin is secreted by the salivary glands. Because there is evidence that some of these striated duct proteins are secreted basally, i.e. into the circulation,^33^-^35^ we concluded that EPACS induces the synthesis of salivary adropin, and the quantity of adropin in the saliva could be useful for early management of EPACS, in conjunction with serum adropin measurement. This research also showed that blood levels of CK-MB and CK increased within 30 to 40 minutes (zero time) after EPACS, peaked at six hours, and started to decrease after 12 hours but remained higher than control levels, even at 48 hours.

In this study, receiver operating characteristic (ROC) curves were used to determine the sensitivity and specificity of serum and saliva adropin levels in EPACS patients. At four hours after ACS, serum adropin exhibited 91.7% sensitivity and 50% specificity at a confidence interval of 95% when the cut-off value was 4.43 ng/ml, while serum troponin I exhibited 100% sensitivity and 100% specificity at a confidence interval of 95% when the cut-off value was 0.141 ng/ml. At four hours after ACS, the saliva adropin concentration had a sensitivity of 91.7% and a specificity of 57% at a confidence interval of 95% when the cut-off value was 4.12 ng/ml. At six hours after ACS, the serum adropin exhibited 91.7% sensitivity and 64% specificity at a confidence interval of 95% when the cut-off value was 5.37 ng/ml, while serum troponin I exhibited 100% sensitivity and 100% specificity at a confidence interval of 95% when the cut-off value was 0.226 ng/ml. At six hours after ACS, the saliva adropin concentration had a sensitivity of 91.7% and a specificity of 57% at a confidence interval of 95% when the cut-off value was 4.24 ng/ml. ROC curve analysis indicated that serum troponin I and adropin concentrations diagnosed EPACS with over 90% sensitivity in emergency, cardiology and cardovascular surgery patients.

Serum and saliva adropin measurements were not as specific as serum troponin I for diagnosing EPACS. Serum troponin I was still superior to serum or saliva adropin, even though there is the advantage of taking a salivary sample, which can be collected without a venous blood sample. Nevertheless, serum or saliva adropin could still be useful in diagnosing EPACS in the future. First-generation ELISA adropin kits, even from the same company, gave variable results, and there was even more variability among kits from different companies. More reproducible and automated adropin measurements could overcome the current shortfall in specificity, since diagnosis of EPACS by adropin is highly sensitive.

Our study had other limitations, especially the small number of patients. Also, this was a single-centre study. The results should be confirmed in a prospective, multicentre study involving more patients. Moreover, adropin is expressed in many tissues, and we did not examine their possible contributions as potential confounders to our measured saliva and serum adropin concentrations. Our previous animal studies revealed that liver and kidney tissue adropin concentrations were considerably changed by isoproterenol-induced EPACS.^19^

The composition and production of saliva is also variable (like urine),36 and this theoretically makes the quantitative determination of any substance unreliable. However, there was a good correlation between the salivary and serum adropin concentrations in this study. This may have been due to the added protease inhibitor (aprotinin) before collection of the biological samples.^25^ Protease inhibitor protects the peptide of interest (adropin) from degradation.^25^ We also believe that a less robust correlation in a larger study could be attributed to this fact.

## Conclusion

Despite these limitations, this study provides novel evidence of a connection between increased saliva/serum adropin levels and EPACS. The saliva adropin concentration was higher than the serum adropin level in subjects with and without EPACS. Adropin is synthesised in the intercalated duct, mucous acinus and interlobular cells of human salivary glands, and this could contribute to the high concentrations in saliva. Saliva adropin levels could also be more useful than serum levels for diagnosing some metabolic conditions.

Saliva offers advantages over blood, including that collection is non-invasive and therefore stress free for patients, especially children,^21^ making it a more suitable choice than serum for measuring adropin in diagnosing EPACS. However there are some limitations and difficulties in using ELISA on saliva, since inhibitors and/or binding proteins could be present and could negatively affect the quantitative determination of peptides/ proteins.

Overall, four and six hours after EPACS, ROC curve analysis demonstrated that the saliva adropin concentration reflected EPACS with 91.7% (at four hours) and 91.7% (at six hours) sensitivity and 57% (at four hours) and 57% (at six hours) specificity. Therefore, four and six hours after EPACS, the saliva adropin concentration showed the same sensitvity and specifity for diagnosing EPACS as the serum level. All these data promise new possibilities for the diagnosis of EPACS, besides measuring other cardiac enzymes and proteins (troponins).
